# Green Biosynthesis of Bioactive Streptomyces Metabolites Using Edible Mushrooms: Implications for Glycemic Control and Hepatic Protection in Diabetic Rats

**DOI:** 10.1002/fsn3.71920

**Published:** 2026-06-05

**Authors:** Yahye Ahmed Nageye, Abdirasak Sharif Ali, Kizito Eneye Bello

**Affiliations:** ^1^ Department of Microbiology and Laboratory Science, Faculty of Medicine and Health Sciences SIMAD University Mogadishu Somalia; ^2^ Department of Microbiology, Faculty of Natural Science Kogi State (Prince Abubakar Audu) University Anyigba Kogi State Nigeria

**Keywords:** edible mushrooms, glycemic control, green biosynthesis, hepatoprotection, *Streptomyces* secondary metabolites

## Abstract

Diabetes mellitus is characterized by chronic hyperglycemia and oxidative stress, frequently leading to hepatic dysfunction. Functional food–based strategies that offer glycemic regulation and organ protection are increasingly sought as safer alternatives to long‐term pharmacotherapy. This study explored the green biosynthesis of *Streptomyces* secondary metabolites using edible mushrooms and evaluated their potential for glycemic control and hepatic protection in diabetic rats. A *Streptomyces* isolate was molecularly identified by 16S rRNA sequencing and cultivated using *Pleurotus ostreatus* extract as a biological mediator for metabolite biosynthesis. Phytochemical composition and antioxidant activity were assessed in vitro. Streptozotocin‐induced diabetic rats were treated orally with biosynthesized metabolite extracts (200 and 400 mg/kg). Fasting blood glucose, liver function enzymes, hepatic oxidative stress markers, histopathology, and molecular docking against diabetes‐related targets were evaluated. The biosynthesized extract contained high levels of total phenolics (68.42 ± 3.15 mg GAE/g) and flavonoids (41.76 ± 2.08 mg QE/g), with strong DPPH scavenging activity (~81%). High‐dose treatment reduced fasting blood glucose from > 300 to 129.6 ± 11.8 mg/dL by Day 28. Serum ALT, AST, and ALP levels were significantly lowered compared to diabetic controls, while hepatic malondialdehyde decreased from 4.92 ± 0.41 to 2.18 ± 0.21 nmol/mg protein. Molecular docking revealed strong binding affinities against α‐glucosidase (−10.9 kcal/mol) and oxidative stress–related proteins (−7.9 kcal/mol). Edible mushroom–mediated biosynthesis of *Streptomyces* metabolites produced a bioactive extract with significant glycemic regulatory, antioxidant, and hepatoprotective effects, supporting its potential application as a functional food or nutraceutical for metabolic health management.

## Graphical abstract

Mushroom‐mediated *Streptomyces* metabolites improved fasting blood glucose, reduced hepatic injury markers, restored antioxidant defenses, and showed supportive molecular docking interactions, indicating potential as a functional food or nutraceutical for diabetes‐associated metabolic and liver protection.

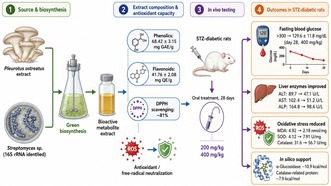

## 1 Introduction

Diabetes mellitus continues to be a significant global public health issue, marked by persistent hyperglycemia and related metabolic disorders that increase the risk of serious consequences, such as hepatic dysfunction (Alam et al. 2021; Antar et al. 2023; Tomic et al. 2022). The liver is crucial for glucose regulation, lipid metabolism, and detoxification; hence, hepatic impairment caused by diabetes markedly deteriorates disease prognosis (Petersen et al. 2017). Although traditional antidiabetic medications are accessible, long‐term therapy frequently encounters limitations due to unwanted effects, elevated costs, and diminished efficacy with time (Cheng et al. 2024). As a result, there is increasing interest in food‐derived bioactive chemicals and nutraceutical approaches that can aid glycemic regulation and save essential organs, such as the liver, through dietary modifications (Lewis Lujan et al. 2024).

Edible mushrooms have historically been utilized as functional foods owing to their nutritional density and health‐enhancing attributes. They are acknowledged sources of dietary fiber, vital amino acids, vitamins, minerals, and a diverse range of bioactive substances, such as polysaccharides, phenolics, terpenoids, and sterols (Dey 2026). Various research has evidenced the antidiabetic, antioxidant, immunomodulatory, and hepatoprotective properties of edible mushrooms, endorsing their incorporation into dietary strategies for metabolic health (Fourier et al. 2021; Ibrahim et al. 2024). In addition to their inherent bioactivity, edible mushrooms have recently attracted interest as sustainable, food‐grade biological platforms for the environmentally friendly biosynthesis of value‐added compounds, due to their enzymatic versatility, safety for human consumption, and compatibility with eco‐friendly production systems (Kumar et al. 2021).

Green biosynthesis utilizing biological systems presents a viable alternative to traditional chemical synthesis, which frequently employs toxic reagents and environmentally detrimental procedures. In food science and nutrition, biologically mediated synthesis is appealing as it adheres to the ideals of sustainability, safety, and customer acceptance. Edible mushrooms serve as an exceptional interface between food matrices and microbial metabolism, facilitating the generation or alteration of bioactive substances for nutraceutical and functional food applications (Abuajah et al. 2014).

Streptomyces species are renowned in microbiology for their exceptional ability to synthesize structurally varied secondary metabolites with extensive biological activity (de Lima Procópio et al. 2012). These metabolites encompass molecules possessing antioxidant, anti‐inflammatory, antibacterial, and metabolic regulatory characteristics (Bello et al. 2024; Chevrette et al. 2019; Salwan and Sharma 2000). Although chemicals originating from Streptomyces have historically been investigated in pharmaceutical research, there is a growing acknowledgment of their potential significance in food and nutrition sciences when generated or processed via food‐grade processes. Interactions between Streptomyces and fungi significantly affect metabolite profiles, augment bioactivity, and facilitate the production of new chemicals via microbial–fungal metabolic crosstalk.

The integration of Streptomyces metabolism with edible mushroom biosynthesis constitutes a novel approach that connects food microbiology and metabolic health studies. This technique facilitates the production of bioactive microbial metabolites within a physiologically safe and nutritionally pertinent framework, hence broadening their utility beyond pharmaceutical contexts to include functional food and nutraceutical development. Nonetheless, despite increasing interest in microbial–fungal biosynthesis, few research have investigated the metabolic health effects of Streptomyces secondary metabolites generated with edible mushrooms as biological mediators. Streptozotocin‐induced diabetic rats are commonly utilized as experimental models to assess the metabolic and organ‐protective properties of food‐derived bioactive. Streptozotocin‐induced diabetes replicates essential characteristics of real diabetes, such as hyperglycemia, oxidative stress, and hepatic damage, rendering it an invaluable model for evaluating glycemic control and liver functionality (Rebecca Roy et al. 2023).

This study examines the green production of Streptomyces secondary metabolites utilizing edible mushrooms as biological mediators and assesses their impact on glycemic regulation and liver protection in streptozotocin‐induced diabetic rat models.

## 2 Materials and Methods

### 2.1 Microbial Strain and Edible Mushroom Material

The Streptomyces isolate utilized in this research was obtained from agricultural soil enriched with animal manure, a habitat known for its abundance of actinomycetes exhibiting varied metabolic functions. Soil samples were collected under aseptic conditions, air‐dried, and subjected to serial dilution prior to inoculation onto starch casein agar augmented with specific antifungal agents to suppress fungal proliferation. Plates were incubated under aerobic conditions, and colonies exhibiting classic Streptomyces traits, such as filamentous growth and an earthy odor, were repeatedly subcultured to achieve pure isolates. The chosen isolate was preserved on starch casein agar slants at 4°C and routinely subcultured to confirm viability (Bello et al. 2024).

The Streptomyces isolate was molecularly identified by 16S rRNA gene sequencing. Genomic DNA was isolated from actively proliferating cells employing a conventional bacterial DNA extraction procedure. The 16S rRNA gene was amplified via polymerase chain reaction (PCR) employing universal bacterial primers (Jose and Jebakumar 2012; Khadayat et al. 2020). PCR results were validated using agarose gel electrophoresis, subsequently purified, and commercially sequenced. The acquired sequence was analyzed against reference sequences in the National Center for Biotechnology Information (NCBI) database utilizing the BLAST tool to ascertain phylogenetic membership. The isolate was characterized by its best sequence similarity to confirmed Streptomyces species, and this sequence was utilized for phylogenetic analysis.

The edible mushroom species *Pleurotus ostreatus* was chosen for its recognized safety for human consumption, nutritional significance, and recorded bioactive characteristics (Yan Zhang et al. 2016). Fresh fruiting bodies were sourced from a certified local producer and verified by a knowledgeable mycologist. The mushrooms were meticulously cleansed with distilled water to eliminate surface impurities, sliced, and air‐dried at room temperature to retain heat‐sensitive chemicals. The desiccated samples were pulverized into a fine powder and preserved in airtight containers at 4°C for subsequent utilization in the green biosynthesis of secondary metabolites.

### 2.2 Green Biosynthesis of Streptomyces Secondary Metabolites

Biosynthesis mediated by edible mushrooms was conducted utilizing an aqueous extract of the mushroom as a biological substrate. Mushroom powder was extracted using distilled water with gentle heating, followed by filtration and sterilization. The extract was integrated into an altered fermentation medium and inoculated with the Streptomyces strain under sterile conditions. Fermentation was performed at regulated temperature and agitation for a specified duration to enhance secondary metabolite synthesis. Upon completion of fermentation, the culture broth was subjected to centrifugation to eliminate biomass, and metabolites were extracted from the supernatant utilizing food‐compatible organic solvents. The extracts were concentrated under decreased pressure and subsequently kept at 4°C until further examination.

### 2.3 Phytochemical and Antioxidant Characterization

A preliminary qualitative assessment of the biosynthesized metabolites was conducted to identify principal bioactive groups, including phenolics, flavonoids, and alkaloid‐like substances. The total phenolic and flavonoid concentrations were measured with conventional colorimetric techniques (Sari et al. 2023). The antioxidant activity was evaluated by recognized in vitro assays, including DPPH radical scavenging and ferric reducing antioxidant power (FRAP), to substantiate the metabolic significance of the extracts (Iqbal et al. 2024).

### 2.4 Experimental Animals and Ethical Approval

Adult male Wistar rats ( *Rattus norvegicus* ) weighing 180–220 g were obtained from a certified animal breeding facility and acclimatized for 7 days prior to experimentation under controlled laboratory conditions (temperature 22°C ± 2°C, relative humidity 50%–60%, and a 12 h light/dark cycle), with free access to standard pellet diet and clean drinking water. All experimental procedures were conducted in accordance with internationally accepted guidelines for the care and use of laboratory animals and adhered to the principles of Replacement, Reduction, and Refinement (3Rs). Ethical approval was obtained from the Institutional Animal Ethics Committee (Approval No. PAUU/CRL/MCB03). Experimental diabetes was induced using a single intraperitoneal injection of streptozotocin (STZ) (55 mg/kg STZ), freshly prepared in 0.1 M citrate buffer (pH 4.5), following the method described by Huang et al. (2024) (Huang et al. 2024). After 72 h, fasting blood glucose levels were measured using a glucometer, and animals with glucose levels ≥ 250 mg/dL were considered diabetic and included in the study. A randomized controlled experimental design was employed, and eligible animals were randomly allocated into groups (*n* = 5 per group) using a simple randomization method. The experimental groups comprised a normal control group (non‐diabetic, vehicle‐treated), a diabetic control group (untreated), a standard reference group receiving a conventional antidiabetic drug, and treatment groups administered mushroom‐mediated *Streptomyces* metabolite extracts at graded doses (100, 200, and 400 mg/kg). All treatments were administered orally once daily for a period of 28 days. Investigators responsible for outcome assessment were blinded to group allocation to minimize bias. The primary outcome measured was fasting blood glucose level, while secondary outcomes included changes in body weight and relevant biochemical parameters assessed at predetermined intervals throughout the study.

### 2.5 Induction of Diabetes and Experimental Design

Diabetes was produced using a single intraperitoneal injection of streptozotocin (STZ) freshly formulated in citrate buffer (pH 4.5) (Huang et al. 2024). Following a 72‐h period, fasting blood glucose levels were assessed, and rats exhibiting verified hyperglycemia were incorporated into the study. Animals were randomly allocated into experimental groups, comprising a normal control group, a diabetic control group, a standard reference group, and treatment groups administered mushroom‐mediated Streptomyces metabolite extracts at varying doses. Treatments were supplied orally for a specified trial duration to correspond with dietary intake significance.

### 2.6 Evaluation of Glycemic and Hepatic Biomarkers

Fasting blood glucose levels were systematically assessed utilizing a glucometer. Upon conclusion of the treatment period, the animals were euthanized, and blood samples were obtained for biochemical analysis (Xia et al. 2019). Serum concentrations of hepatic function enzymes, specifically alanine aminotransferase (ALT), aspartate aminotransferase (AST), and alkaline phosphatase (ALP), were assessed with commercial diagnostic kits (Suksri et al. 2025). Hepatic tissue was removed, homogenized, and examined for oxidative stress markers, including malondialdehyde (MDA), superoxide dismutase (SOD), and catalase activity (Hafez et al. 2024).

### 2.7 Histopathological Examination

Liver tissues were preserved in neutral buffered formalin, subjected to conventional histological methods, sectioned, and stained with hematoxylin and eosin. Microscopic examination of sections was conducted to identify structural abnormalities, such as hepatocellular degeneration, inflammatory infiltration, and sinusoidal distortion (Dunn et al. 2024).

### 2.8 Molecular Docking Analysis

In order to substantiate the biological findings and investigate potential mechanisms of action, in silico molecular docking was conducted. Key discovered or representative molecules from the biosynthesized metabolites were chosen as ligands. Three‐dimensional structures of target proteins pertinent to glucose metabolism and hepatic function, including α‐glucosidase and essential oxidative stress–related enzymes, were obtained from the Protein Data Bank. Ligand structures were refined, and docking simulations were performed utilizing AutoDock Vina. Binding affinities, interaction energies, and critical amino acid interactions were examined and illustrated to forecast ligand–protein binding stability and biological significance (Wang et al. 2023).

### 2.9 Statistical Analysis

Data were presented as mean ± standard deviation. Statistical comparisons between groups were conducted using one‐way analysis of variance (ANOVA) accompanied by suitable post hoc tests. Statistical significance was established at *p* < 0.05.

## 3 Results

### 3.1 Molecular Identification of the *Streptomyces* Isolate

**FIGURE 1:**
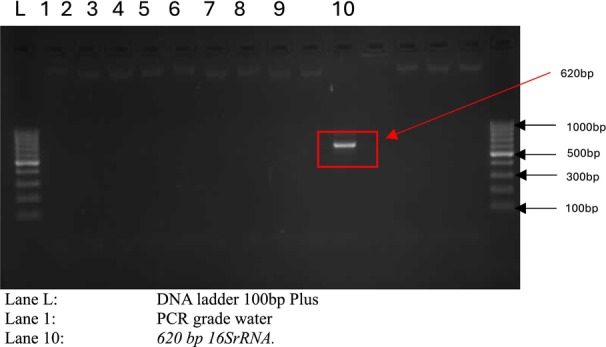
Agarose gel electrophoresis of PCR‐amplified 16S rRNA gene of the *Streptomyces* isolate.

**FIGURE 2:**
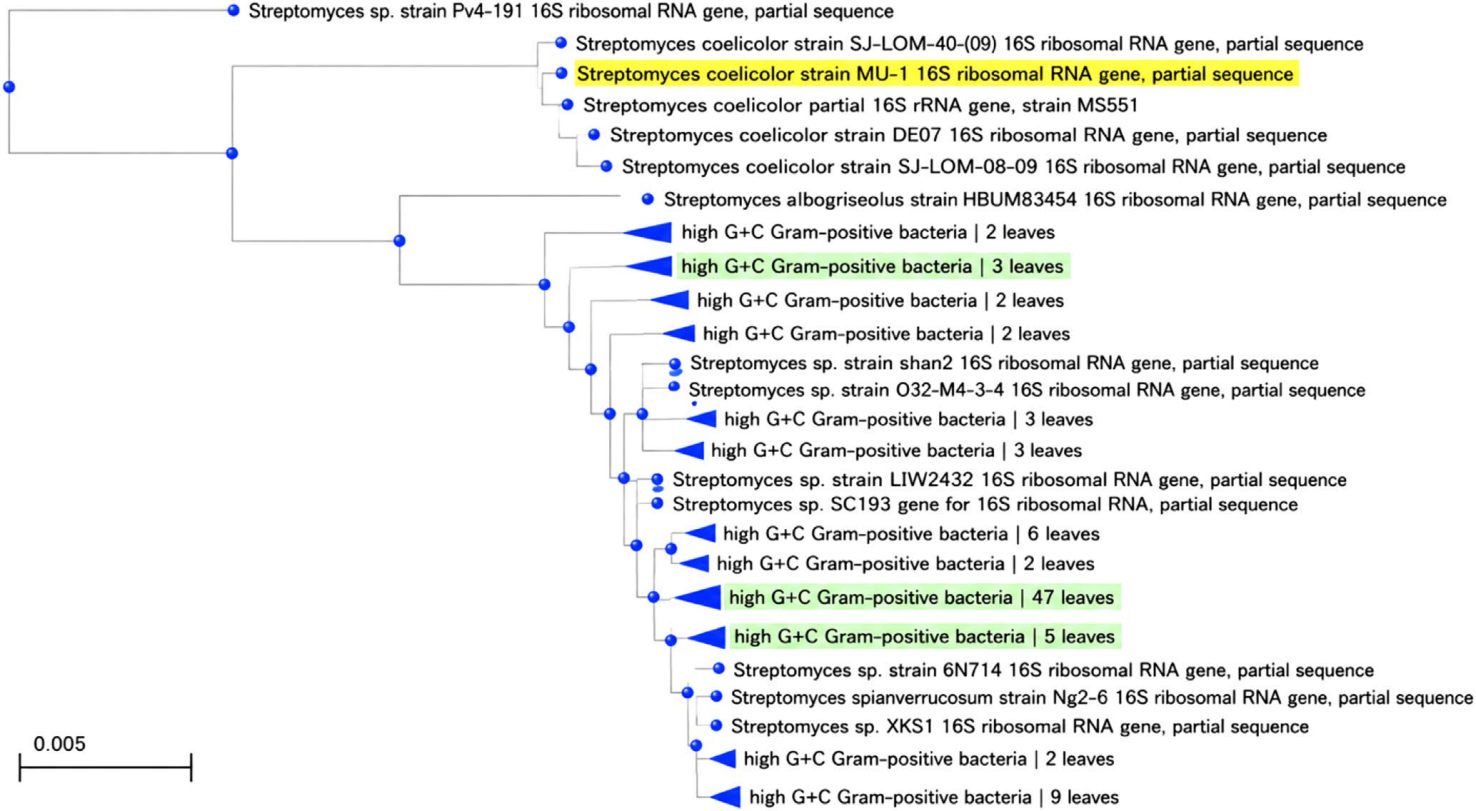
Phylogenetic tree based on 16S rRNA gene sequences showing the relationship between the isolated *Streptomyces* strain and reference species. The organism highlighted in yellow is the queried isolate.

Precise identification of the microbial isolate is crucial for ensuring repeatability and contextualizing its metabolic capabilities. The 16S rRNA gene sequencing results verified that the isolate obtained from animal‐manured soil is classified under the genus Streptomyces (Figure 1), a group renowned for its metabolic flexibility. The significant sequence similarity (≥ 98%) with reference Streptomyces species and its distinct clustering within a specific phylogenetic clade confirm the taxonomic identity of the isolate utilized for edible mushroom‐mediated biosynthesis (Figure 2).

### 3.2 Phytochemical Composition of Biosynthesized Metabolites

The quantitative phytochemical study revealed that the Streptomyces metabolite extract, mediated by mushrooms, had significant concentrations of phenolic and flavonoid components. The overall phenolic content (68.42 ± 3.15 mg GAE/g extract) and flavonoid content (41.76 ± 2.08 mg QE/g extract) (Table 1) signify a substantial presence of redox‐active compounds. The values align with those documented for functional food and nutraceutical extracts, indicating that the biosynthetic process produced chemicals potentially significant for metabolic health modulation.

**TABLE 1:** Phytochemical composition of edible mushroom–mediated *Streptomyces* secondary metabolite extract.

Parameter	Value (mean ± SD)
Total phenolic content (mg GAE/g extract)	68.42 ± 3.15
Total flavonoid content (mg QE/g extract)	41.76 ± 2.08

### 3.3 In Vitro Antioxidant Activity

**FIGURE 3:**
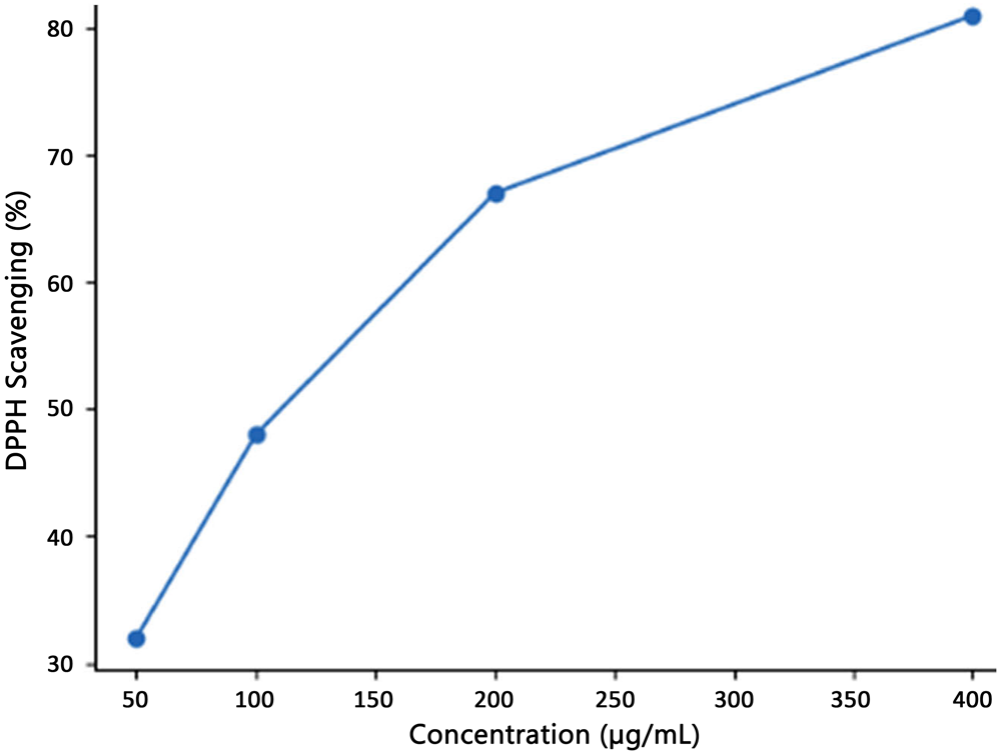
DPPH radical scavenging activity of edible mushroom–mediated *Streptomyces* metabolite extract.

**FIGURE 4:**
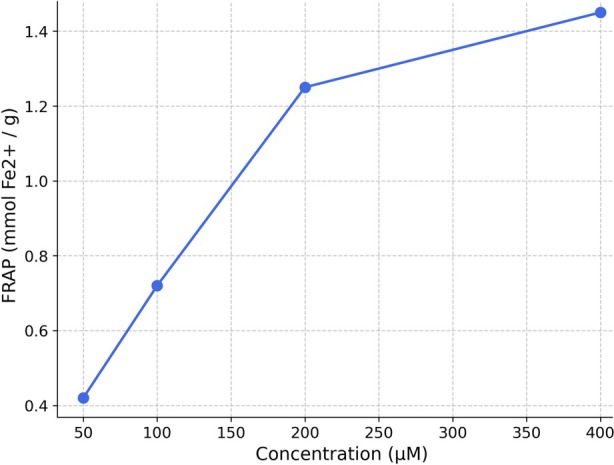
Ferric reducing antioxidant power (FRAP) of biosynthesized *Streptomyces* metabolites.

The antioxidant activity of the biosynthesized metabolites was assessed to determine their functional significance in relation to oxidative stress‐related metabolic diseases. The extract demonstrated significant, concentration‐dependent DPPH radical scavenging activity and ferric reducing power (Figures 3 and 4), indicating its capacity to donate electrons and neutralize free radicals.

### 3.4 Effect on Fasting Blood Glucose Levels

The administration of streptozotocin to induce diabetes led to significant hyperglycemia in untreated diabetic rats, with fasting blood glucose levels consistently surpassing 300 mg/dL during the trial duration. The oral administration of the mushroom‐derived Streptomyces metabolite extract resulted in a significant and dose‐dependent decrease in fasting blood glucose levels. On Day 28, the high‐dose treatment group (400 mg/kg) attained glucose levels (129.6 ± 11.8 mg/dL) (Table 2) similar to those of the standard metformin‐treated group, demonstrating a significant glycemic regulatory effect without full normalization, consistent with nutraceutical interventions.

**TABLE 2:** Effect of biosynthesized *Streptomyces* metabolites on fasting blood glucose levels (mg/dL).

Group	Day 0	Day 14	Day 28
Normal control	92.4 ± 6.3	94.1 ± 5.8	93.7 ± 6.1
Diabetic control	312.6 ± 18.9	325.4 ± 21.3	338.2 ± 24.6
Standard drug (Metformin)	305.8 ± 17.6	176.3 ± 14.2*	118.7 ± 10.9*
Low‐dose extract (200 mg/kg)	308.4 ± 19.2	221.6 ± 16.4*	164.9 ± 13.7*
High‐dose extract (400 mg/kg)	310.1 ± 20.1	189.8 ± 15.1*	129.6 ± 11.8*

* *p* < 0.05 vs. diabetic control.

### 3.5 Liver Function Biomarkers

Analysis of serum liver enzymes indicated significant hepatic damage in diabetic control rats, as demonstrated by increased levels of ALT, AST, and ALP (Table 3). The treatment with the biosynthesized metabolite extract markedly reduced these elevations in a dose‐dependent fashion. The high‐dose group exhibited enzyme levels nearing normal control values, indicating successful maintenance of hepatic integrity. The results suggest that the extract alleviated liver impairment associated with diabetes rather than producing a pharmaceutical normalizing effect.

**TABLE 3:** Effect of biosynthesized metabolites on liver function enzymes.

Parameter	Normal	Diabetic	Standard	Low dose	High dose
ALT (U/L)	38.6 ± 3.4	89.7 ± 6.8	44.3 ± 4.1^*^	56.8 ± 5.2^*^	47.1 ± 4.3^*^
AST (U/L)	42.1 ± 3.9	102.4 ± 7.6	49.6 ± 4.7^*^	61.3 ± 5.9^*^	51.2 ± 4.8^*^
ALP (U/L)	81.5 ± 6.2	164.8 ± 12.3	92.7 ± 7.1^*^	114.5 ± 9.6^*^	98.6 ± 7.9^*^

^*^ Significance at 0.05 probability value at 95% confidence interval.

### 3.6 Oxidative Stress Markers in Liver Tissue

Oxidative stress generated by diabetes was indicated by increased hepatic malondialdehyde levels and diminished activity of endogenous antioxidant enzymes in diabetic control subjects (Table 4). The administration of the biosynthesized metabolite extract markedly reduced lipid peroxidation and reinstated superoxide dismutase and catalase activity. The significant restoration of antioxidant defenses, especially in the high‐dose group, indicates that the extract efficiently regulated oxidative equilibrium in liver tissue.

**TABLE 4:** Hepatic oxidative stress markers.

Parameter	Normal	Diabetic	Low dose	High dose
MDA (nmol/mg protein)	1.84 ± 0.16	4.92 ± 0.41	3.12 ± 0.28^*^	2.18 ± 0.21^*^
SOD (U/mg protein)	8.96 ± 0.74	4.12 ± 0.39	6.58 ± 0.52^*^	7.91 ± 0.63^*^
Catalase (U/mg protein)	62.4 ± 4.9	31.6 ± 3.1	48.9 ± 4.3^*^	56.7 ± 4.8^*^

^*^ Significance at 0.05 probability value at 95% confidence interval.

**FIGURE 5:**
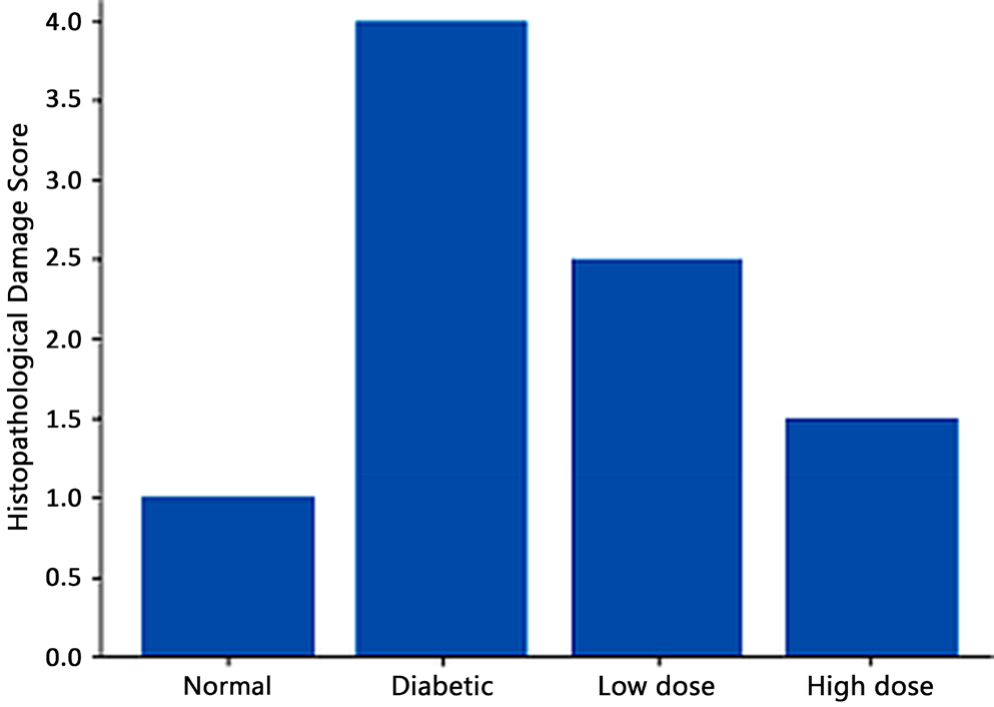
Semi‐quantitative assessment of hepatic histopathological damage in experimental groups.

**FIGURE 6:**
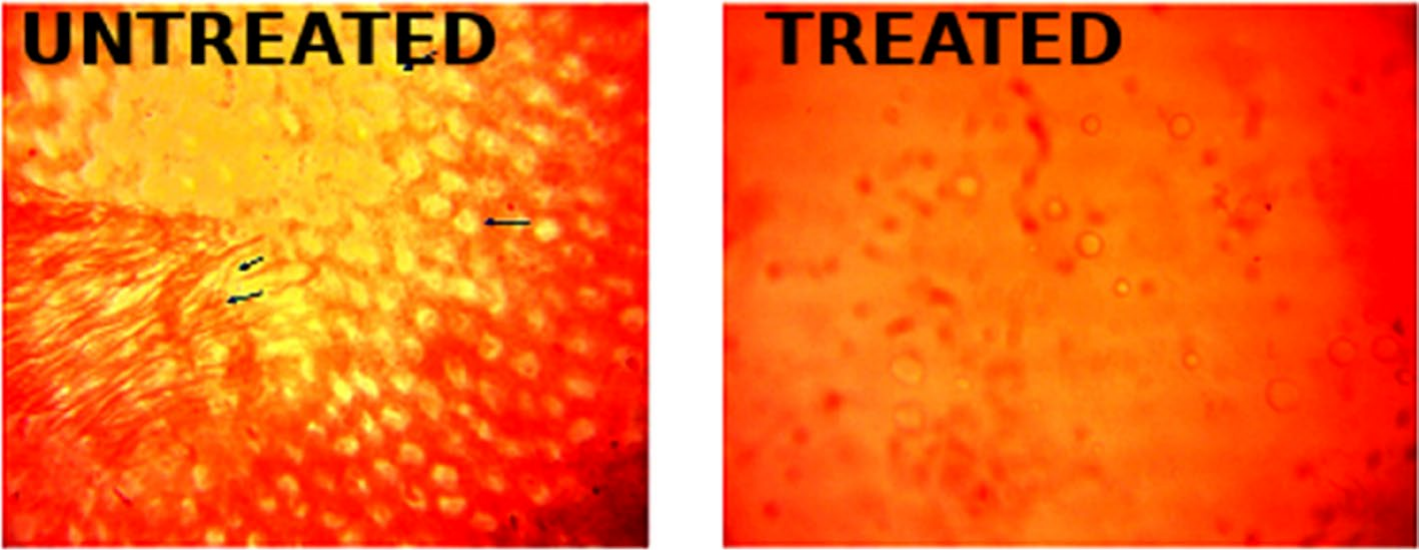
Histopathological examination of liver tissue. Arrows pointing at liver scarring cells and inflammation in the untreated rat. Representative photomicrographs of liver sections showing histopathological changes following diabetic induction and treatment. Diabetic untreated group exhibits marked hepatocellular degeneration, pronounced inflammatory cell infiltration, and indicates severe hepatic injury. Treatment group shows substantial preservation of hepatic architecture with intact hepatocytes and reduced inflammatory infiltration. Scale bar = 50 μm.

Histopathological analysis of liver sections demonstrated significant structural changes indicative of diabetes‐induced hepatic damage. In the untreated diabetes cohort, the hepatic architecture was significantly compromised, marked by hepatocellular degeneration, which included cytoplasmic vacuolation and the obliteration of normal cellular borders (Figures 5 and 6). These alterations were accompanied by significant infiltration of inflammatory cells, especially in the periportal region, indicating an active inflammatory response.

Conversely, liver slices from the treated groups exhibited a relative preservation of hepatic architecture, characterized by less hepatocellular degeneration and a significant reduction in inflammatory infiltration. Hepatocytes exhibited greater organization surrounding the major vein, and the sinusoidal gaps were relatively recovered. The histological enhancements indicate a protective and restorative influence of the biosynthesized Streptomyces metabolites on hepatic tissue, perhaps facilitated by enhanced glycemic regulation and reduction of oxidative stress.

### 3.7 Molecular Docking Analysis

**FIGURE 7:**
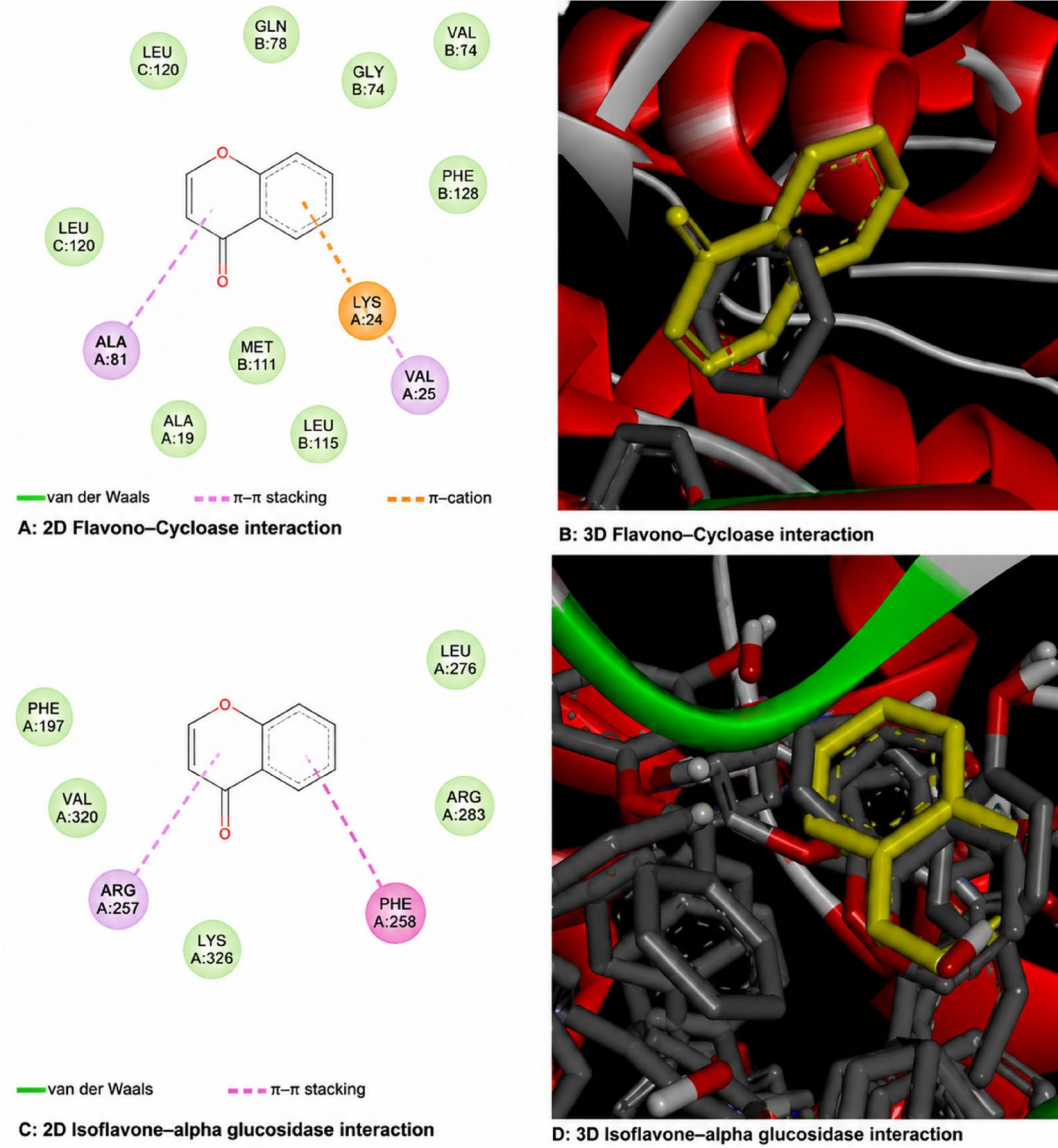
Ligand‐receptor interactions of the extract key bioactive compounds against diabetes‐related proteins.

Molecular docking analysis was conducted to offer mechanistic evidence for the reported biological effects. The chosen biosynthesized compounds had advantageous binding affinities for α‐glucosidase and oxidative stress‐related target proteins, with binding energies between −7.9 and −10.9 kcal/mol (Table 5). These interaction profiles indicate permanent ligand–protein interactions and potential modification of enzyme activity, corroborating the in vivo glycemic and hepatoprotective results found in this work (Figure 7).

**TABLE 5:** Binding energies of selected biosynthesized compounds against metabolic targets.

Compound	Target protein	Binding energy (kcal/mol)
Compound A: Amentoflavone (1617‐53‐4‐Didemethyl‐ginkgetin‐3′,8″‐Biapigenin‐Tridemethylsciadopitysin)	α‐Glucosidase	−10.9
Compound B: Flavone (2‐Phenyl‐4H‐chromen‐4‐one)	Catalase‐related protein	−7.9

## 4 Discussion

This study revealed that the manufacturing of Streptomyces secondary metabolites facilitated by edible mushrooms yielded a physiologically active extract that exhibited substantial antioxidant, glycemic regulating, and hepatoprotective properties in a streptozotocin (STZ) induced diabetic rat model. This research, framed within food science and nutrition, substantiates the notion of employing food‐compatible microbial‐fungal bioprocesses to produce functional chemicals with prospective applications in nutraceutical and dietary approaches for metabolic health.

The biosynthesized extract exhibited significant concentrations of total phenolics and flavonoids, chemicals typically linked to antioxidant and health‐enhancing properties in food (Shahidi and Ambigaipalan 2015; Zhang et al. 2011). Phenolic and flavonoid compounds have been extensively documented in edible mushrooms and associated with their free radical scavenging and redox modulation abilities, rendering them significant functional elements in food science research (Reis et al. 2017; Valverde et al. 2015). In accordance with these findings, the extract demonstrated significant DPPH radical scavenging activity and enhanced ferric reducing antioxidant capacity with increasing concentration.

Antioxidant activity is especially pertinent to metabolic diseases, since oxidative stress leads to β cell malfunction, insulin resistance, and organ damage (Heleno et al. 2012). Multiple studies indicate that mushroom extracts abundant in phenolics and polysaccharides might augment antioxidant levels in diabetic animals, diminishing lipid peroxidation and enhancing endogenous defenses like superoxide dismutase (SOD) and catalase (Chun et al. 2021; Hamzah et al. 2023; Rani et al. 2016). The functional antioxidant capabilities identified here correspond with research that supports the significance of mushroom‐derived substances as dietary antioxidants in a nutritional framework.

STZ treatment resulted in sustained hyperglycemia in untreated control rats (> 300 mg/dL), aligning with standard diabetic rodent models (Chin et al. 2019). The oral delivery of the mushroom‐mediated Streptomyces extract led to a considerable and dose‐dependent decrease in fasting blood glucose levels. By Day 28, the high‐dose extract (400 mg/kg) lowered glucose levels to approximately 129.6 mg/dL, nearly aligning with the glucose levels seen with regular metformin therapy.

Mushroom‐derived functional foods have demonstrated efficacy in enhancing glycemic control in diabetes animals, primarily via polysaccharides or secondary metabolites that influence glucose metabolism and insulin signaling (Ibrahim et al. 2024; Shamim et al. 2023). For instance, ß‐glucans derived from Pleurotus species have been documented to enhance glucose tolerance and insulin sensitivity by modulating glucose transporter expression and glycolytic enzyme activity (Ibrahim et al. 2024). The extract utilized in this study, generated through edible mushroom‐mediated biosynthesis of microbial metabolites, likely exhibits a comparable array of bioactive effects that promote moderate glycemic regulation, aligning with definitions of functional foods that positively impact physiological functions beyond fundamental nutrition (Reis et al. 2017).

The liver is pivotal in glucose and lipid metabolism and is susceptible to oxidative damage in diabetes. Increased blood concentrations of ALT, AST, and ALP in diabetic control rats validated hepatic damage associated with diabetes. The metabolite extract treatment markedly reduced these enzyme increases, with the high‐dose group exhibiting enzyme levels nearing those of normal controls. The biochemical results were validated by histological findings demonstrating enhanced preservation of liver architecture.

Components of edible mushrooms, specifically phenolics and polysaccharides, have been documented to provide hepatoprotective benefits in preclinical models, frequently associated with their antioxidant and anti‐inflammatory characteristics (Abdelkader et al. 2025; Acosta‐Urdapilleta et al. 2020; Jovanović et al. 2021). In functional food research, such benefits underscore the potential of particular food components to improve organ dysfunction linked to chronic metabolic stress, hence endorsing their incorporation into dietary regimens for managing metabolic health.

STZ‐induced oxidative stress was demonstrated by increased hepatic malondialdehyde (MDA) levels and reduced activity of endogenous antioxidants such as SOD and catalase. The administration of the mushroom‐mediated extract dramatically reduced MDA levels and enhanced antioxidant enzyme activity in a dose‐dependent manner, indicating a reduction in oxidative damage to hepatic tissue.

Comparable antioxidant recoveries have been documented with mushroom polysaccharides and phenolic extracts in models of diabetic and chemically induced liver injury (Abdelkader et al. 2025; Mishra et al. 2013; Wang et al. 2022). These findings underscore the importance of antioxidant functional foods in alleviating oxidative stress, highlighting the nutritional value of redox‐active chemicals in food matrices and their role in maintaining tissue integrity during metabolic stress.

Molecular docking simulations were conducted with chosen bioactive compounds to complement the in vivo results against major metabolic targets. Binding energies between −7.9 and −10.9 kcal/mol for proteins like α‐glucosidase and catalase indicate potential interactions that may explain the functional benefits seen in glycemic regulation and oxidative stress reduction.

Although docking findings are predictive and necessitate specific validation via biochemical assays, they offer insights into potential molecular pathways pertinent to functional food research. Inhibition of carbohydrate digesting enzymes, such as α‐glucosidase regulation, is a proven method for diminishing postprandial glucose fluctuations and is employed in dietary strategies to enhance glycemic control (Wang et al. 2023). The docking results provide corroborative evidence that chemicals in the biosynthesized extract may enhance its functional benefits.

The notion of functional foods includes food components that offer health advantages beyond fundamental nutrition, such as the control of metabolic processes and the mitigation of disease (Abuajah et al. 2014; Dey 2026). Mushrooms have been acknowledged as potential functional foods because of their abundant polysaccharides, phenolics, vitamins, and minerals, as well as their established effects on immune function, lipid metabolism, and glucose homeostasis (Dey 2026; Reis et al. 2017). This study advances the paradigm by utilizing an edible mushroom substrate to facilitate the manufacture of microbial secondary metabolites, resulting in a complex extract whose biological effects correspond with functional food aims.

In contrast to pharmaceutical therapies that seek optimal therapeutic outcomes, functional foods strive for moderate, persistent physiological regulation that can be seamlessly incorporated into everyday dietary habits with minimum deleterious consequences (Reis et al. 2017). The modest decreases in glucose levels, enhanced antioxidant status, and hepatoprotective tendencies reported herein substantiate the designation of this biosynthesized extract as a potential functional food component or nutraceutical supplement rather than a pharmaceutical drug.

This study indicates that microbial metabolite extracts derived from edible mushrooms may operate as beneficial food elements for health in metabolic illnesses like diabetes. Integrating these extracts into food matrices or dietary supplements may offer consumers biologically active compounds that enhance glucose management, bolster antioxidant defenses, and promote liver health within comprehensive nutritional regimens.

Due to the escalating global incidence of type 2 diabetes and metabolic syndrome, there is growing interest in dietary strategies that provide metabolic advantages without the adverse effects linked to pharmacological treatments (Corkey 2012; Draznin et al. 2022). Edible mushrooms are often classified as generally recognized as safe (GRAS) and are extensively consumed, rendering them appealing substrates for the production of value‐added functional compounds by microbial biosynthetic methods. This microbial‐fungal interaction enhances the array of food‐compatible procedures employed in the development of next‐generation nutraceuticals.

## 5 Conclusion

The manufacture of Streptomyces secondary metabolites facilitated by edible mushrooms produced a complex extract exhibiting significant antioxidant, glycemic regulating, and hepatoprotective properties in a STZ‐induced diabetic rat model. These effects were corroborated by biochemical, histological, and in silico data, and are consistent with the functional food paradigm by illustrating health‐related advantages beyond fundamental nutrition.

## Author Contributions

**Yahye Ahmed Nageye:** conceptualization, writing – original draft. **Kizito Eneye Bello:** conceptualization, methodology, formal analysis, writing – review and editing, writing – original draft, data curation. **Abdirasak Sharif Ali:** writing – original draft, writing – review and editing, methodology.

## Funding

This study received grants from the Center for Research and Development, SIMAD University.

## Ethics Statement

All experimental procedures involving animals were reviewed and approved by the Institutional Animal Ethics Committee (IAEC), under approval number of PAUU/CRL/MCB03. The study was conducted in strict accordance with internationally accepted guidelines for the care and use of laboratory animals, and all efforts were made to minimize animal suffering and reduce the number of animals used, in line with the principles of Replacement, Reduction, and Refinement (3Rs).

## Conflicts of Interest

The authors declare no conflicts of interest.

## Data Availability

Data sharing not applicable to this article as no datasets were generated or analysed during the current study.
